# P2X_7_ Receptor and APOE Polymorphisms and Survival from Heart Failure: A Prospective Study in Frail Patients in a Geriatric Unit

**DOI:** 10.14336/AD.2016.1202

**Published:** 2017-07-21

**Authors:** Giuseppe Pasqualetti, Marta Seghieri, Eleonora Santini, Chiara Rossi, Edoardo Vitolo, Livia Giannini, Maria Giovanna Malatesta, Valeria Calsolaro, Fabio Monzani, Anna Solini

**Affiliations:** Department of Clinical and Experimental Medicine University of Pisa, Pisa, Italy; Department of Clinical and Experimental Medicine University of Pisa, Pisa, Italy; Department of Clinical and Experimental Medicine University of Pisa, Pisa, Italy; Department of Clinical and Experimental Medicine University of Pisa, Pisa, Italy; Department of Clinical and Experimental Medicine University of Pisa, Pisa, Italy; Department of Clinical and Experimental Medicine University of Pisa, Pisa, Italy; Department of Clinical and Experimental Medicine University of Pisa, Pisa, Italy; Department of Clinical and Experimental Medicine University of Pisa, Pisa, Italy; Department of Clinical and Experimental Medicine University of Pisa, Pisa, Italy; Department of Clinical and Experimental Medicine University of Pisa, Pisa, Italy

**Keywords:** heart failure, brain natriuretic peptide, P2X_7_ receptor, apolipoprotein E, polymorphisms, long-term mortality

## Abstract

Heart failure (HF) is one of the most frequent cause of hospitalization in elderly and often coexists with concurrent geriatric syndromes, like cognitive disturbances; various pathophysiological mechanisms are shared by HF and cognitive decline, notably a substrate of low-grade inflammation. We investigated whether SNPs in the purinergic receptor (P2X_7_R) and apolipoprotein (APO) E genes, both involved in a series of inflammatory responses, are associated to HF or cognitive impairment and are able to predict post-discharge mortality in the elderly. We prospectively analyzed 198 patients (age 85 ± 8 years, predominantly females) admitted to a Geriatric unit for acute HF, whose diagnosis was based on clinical signs, brain natriuretic peptide (BNP) values and ecocardiography in uncertain diagnosis (BNP values between 100 and 400 pg/mL); cognitive performance was assesed by Short Portable Mental Status Questionnaire (SPMSQ). In all the participants, SNPs rs208294 and rs3751143 for P2X_7_R gene and rs429558 and rs7412 for APOE gene were assessed. Information on all-cause mortality was adjudicated by medical records review 36 months after discharge. We found no relationship between P2X_7_R and APOE polymorphisms and 36-month post-discharge mortality; a better outcome for overall survival was observed in patients with BNP values below the median (281 pg/mL) (*p*=0.002) persisting after adjustment for renal function and age, and in those with cognitive impairment (*p*<0.001). Patients harboring APOE-ε4 genotype showed higher BNP concentrations than noncarriers (1289.9 ± 226.9 *vs* 580.5 ± 90.2 pg/mL respectively,*p*=0.004), whereas none of the studied SNPs were associated to impairment in cognitive performance. In conclusion, neither P2X_7_R or APOE genotype seem to predict long-term mortality in elderly patients. Interestingly, APOE-ε4 genotype was associated to higher BNP values, suggesting a putative interaction between genetic and biochemical markers in identifying people at risk for HF.

Acute cardiac diseases, including heart failure (HF), are among the main determinants of mortality in aging individuals; their prevalence dramatically rises in frail individuals with concomitant geriatric syndromes, in particular cognitive disorders, associated with increases in mortality risk, hospital stay, and costs [1, 2]. In aging subjects, HF and cognitive decline, even being fully diverse clinical conditions, might share common mechanisms, with low-grade inflammation playing a role in the pathogenesis of both these diseases [3, 4].

The extracellular signalling molecule (eATP) *via* specific P2 ionotropic (P2X) and metabotropic (P2Y) subtypes receptors is a major mediator of inflammatory responses [5]. The purinergic system is involved in the pathogenesis of cognitive decline, mediating the neuroinflammatory response; a main role is attributed to the P2X_7_ receptor (P2X_7_R), which promotes neuronal death through an activation of microglia-derived IL-1β release [6]. This receptor is highly polymorphic, but not many variants influence its function; the most studied SNPs are 489C>T (*H155Y*) and 1513A>C (*E496A*), linked to a receptor gain-of-function and a loss-of function, respectively [7, 8].

The involvement of purinergic system in myocardial fibrosis and remodeling is well documented [9]; moreover, in ischaemia-reperfusion injury, increased caspase-activity and cell apoptosis are associated to an increased P2X_7_R expression, and P2X_7_R silencing through siRNA, or prevention of the inflammasome assembly by pharmacologic P2X_7_R inhibition, reduce the infarct size in a murine model [10]. In the remodeling process occurring in HF, inflammatory reaction and fibrogenesis are markedly stimulated by IL-1β, whose release depends upon the P2X_7_R-NLRP3 activated inflammasome through the recruitment of pro-inflammatory leukocytes and myocardial fibroblasts [11, 12].

Elevated B-type natriuretic peptide (BNP) levels directly correlate with prognosis, NYHA score, intra-ventricular pressure, pulmonary pressure, and inversely to cardiac output [13], thus representing the most common biomarker of HF, widely used in the clinical practice. It is interesting to point out as BNP promotes myocardial cell apoptosis *via* caspase-1/IL-1β cascade [14]. IL-18 is another cytokine whose release is strictly depending upon P2X_7_R-inflammasome [15]; in subjects carrying HF, IL-18 concentrations are significantly higher compared to healthy controls, although no association was found with functional polymorphisms in P2X_7_R gene [16].

Apolipoprotein E (ApoE) - initially described for its role in lipid homeostasis -is now emerging as a significant risk factor for both cardiovascular and neurologic pathologies [17]. The conformational change typically caused by two SNPs in APOE genes (rs429558 and rs7412) comes out in protein isoforms with critical modifications in protein functionality. These changes can result in variants with broad deleterious effects, like increased cholesterol and triglycerides levels, contributing to a pro-inflammatory milieu and to alter cell signaling pathways [18]. APOE-ε4 has been linked with about 65-75% of sporadic Alzheimer’s disease, but such association is also described for up to 20% of other types of dementia [19]; on the other hand, the link between APOE-ε4 and HF is not strictly consistent, depending firstly on the severity of cardiac disease or the selected population study (low or high risk subjects) [20, 21].

To investigate whether P2X_7_R and APOE polymorphisms impact on long-term mortality in a cohort of older patients with acute HF, and to evaluate their association with concurrent morbidities worsening their prognosis, like cognitive impairment, we designed the present study.

## MATERIAL AND METHODS

### Study population

Two hundred forty-four aging patients were recruited among those consecutively admitted to the Geriatric Unit of University Hospital of Pisa in 2012-2013. Inclusion criteria were age ≥65 years and diagnostic suspect of HF, according to the clinical evaluation in the emergency room; patients evolving into a terminal status within the first 24 hours after admission, or lacking a confirmed diagnosis of HF were then excluded. The study procedures were approved by the Institutional Ethics Committee of Pisa University (n. 3641/2012).

The diagnosis of HF was based on clinical data (personal history of cardiovascular diseases, suggestive signs and symptoms, NYHA score [22]) and the presence of BNP value higher than 100 pg/mL. An electrocardiogram was performed to exclude an acute ischaemic heart disease, while chest radiography allowed differentiating pulmonary affections. In patients falling within an intermediate zone (BNP 100-400 pg/mL), an ultrasonographic measurement of ejection fraction was performed to confirm the diagnosis of systolic dysfunction [23, 24]. According to this diagnostic algorithm, a total of 198 patients participated in the study.

At the admission, a physician specialist in geriatric medicine recorded all the relevant information related to the patient’s acute clinical conditions, medical history, and functional, mental, and sociodemographic status through personal interviews and medical record review. Comorbidity was measured with the Charlson comorbidity index. Baseline functional status was evaluated as the ability to perform six basic activities of daily living (ADLs)-bathing, dressing, transferring, toileting, continence, and feeding-2 weeks before their admission. Cognitive decline was evaluated using the Short Portable Mental Status Questionnaire (SPMSQ) [25], whose score is based on the total number of errors in answering to 10 questions. A diagnosis of cognitive impairment was established when the number of errors was ≥3 (cut-off adjusted by patient’s education level).

### Diagnostic tests

Blood samples were collected from an antecubital vein to extract genomic DNA and to determine blood count and routine analysis. BNP was measured by immunoassay using the ADVIA Centaur System (Bayer Health Care, Tarrytown, NY,USA).

### Genotyping analyses

Blood samples (3 ml) were collected at admission in EDTA tubes and stored at -80°C. DNA extraction was performed using QIAamp DNA Blood Mini Kit (Qiagen, Valencia, CA, USA). Allelic discrimination of genes was performed using an Eco Real-Time System (Illumina Inc., San Diego, CA, USA) according to the standard protocol and with validated TaqMan® SNP genotyping assays (Applied Biosystems Carlsbad, CA, USA). PCR reactions were carried out according to the manufacturer’s protocol. The following polymorphisms were determined: rs7412 and rs429358 for APOE gene (*APOE*) and rs208294 and rs3751143 for P2X_7_R gene (*P2X7R*).

### SNP selection

The SNPs included in our study were selected on the basis of the following reasons: *i)* they modulate gene expression of multifunctional proteins involved in inflammatory response, thus playing a potential role in the pro-atherosclerotic process [26-28] associated with an increased cardiac or neurologic risk profile; *ii)* rs208294 and rs3751143 represent the most common gain and loss of function SNPs of P2X_7_R gene, respectively; *iii)* allelic variations in *APOE* are mainly defined by these two SNPs rs429358 and rs7412; participants with 1 or more copies of ε4 allele (ε2/4 excluded) were considered APOE-ε4 carriers.

#### Follow up

Information on all-cause mortality was checked and completed by medical records review and official information from the national health system 36 months after hospital discharge.

### Statistical analysis

Data were given as mean±SD or SEM, or median [interquartile range]. All polymorphisms were analyzed for deviation from the HWE by *Χ^2^*test. Clinical parameters among the genotypes were evaluated with a one-way analysis of variance (ANOVA) or multivariate analysis of variance (MANOVA) test for continuous variables. Survival curves were calculated by the Kaplan Meier method. Cox Regression was used to obtain an adjusted model. Statistics were performed using JMP?7.0; a *p* value≤0.05 was considered statistically significant.

## RESULTS

### Baseline

Clinical and biochemical characteristics of the whole study group, consisting of 198 patients, are reported in Table 1A and Table 1B, respectively. Mean age was 85 ± 8 years and 61 (32%) were male. Cognitive impairment was strongly represented, being observed in half the cohort; patients suffered of numerous comorbidities and were severely functionally impaired. The majority showed a moderate grade of clinical symptoms (dyspnea) and admission BNP values were nearly three times above the established cut-off for the diagnosis (100 pg/mL).

**Table 1A:** Baseline characteristics of the study group (n=198).

Age (years)	85 ± 8
Sex (M/F)	61/137
BMI (kg/m^2^)	24.7 ± 4.5
SBP (mmHg)	136.7 ± 21.7
DBP (mmHg)	74.8 ± 12.4
Comorbidity (Charlson Index)	7.8 ± 2.0
Severe dependence (ADL)	121 (61.2)
Cognitive impairment (SPMSQ)	100 (50.6)
NYHA functional class	
I	53 (27)
II	89 (45)
III	54 (27)
IV	2 (1)
Medications, n (%)	
RAAS inhibitors	109 (55)
Beta blockers	75 (38)
Diuretics	48 (24)
Anti-platelets	46 (23)

Data are mean±SD or n (%)

Suppl Table A shows as none of the genotypes of the examined polymorphisms significantly deviated from HWE distribution. APOE-ε4 genotype was present in 18% of the cohort (all carriers were heterozygous).

**Table 1B:** Biochemical variables of the study group (n=198).

Red blood cell count (x10^6^/mmc)	3.93 ± 0.92
Hemoglobin (g/dL)	11.1 ± 3.0
White blood cell count (x10^3^/mmc)	7.8 ± 1.6
Platelets (x10^3^/mmc)	232 (130)
Fasting plasma glucose (mg/dL)	115.7 ± 23.6
Total cholesterol (mg/dL)	163.7 ± 13.7
Triglycerides (mg/dL)	124.7 ± 33.5
Fibrinogen (mg/dL)	319 (178)
Creatinine (mg/dL)	1.15 ± 0.63
Estimated glomerular filtration rate (by MDRD, mL/min/1.73m^2^)	58 ± 37
Hs-PCR (mg/dL)	4.66 (8.63)
BNP (pg/mL)	281 (689)

Data are mean±SD or median (interquartile range)

When we analysed whether or not the P2X7R gain-of-function (His155Tyr) or loss-of-function (Glu496Ala) polymorphisms were related with plasma BNP levels, no significant difference emerged between carriers and non carriers of the mutant allele for both polymorphisms. Conversely, APOE-ε4 carriers showed a significantly higher BNP concentrations than noncarriers (Table 2A). In a multivariate model, controlling for age, sex, Charlson comorbidity index and e-GFR, plasma BNP levels were still significantly associated with the presence of APOE-ε4 carriers (*p*=0.03).

Cognitive impairment, according to SPMSQ evaluation, was not significantly related to P2X_7_R genotypes, whilst it trended to be positively linked to the presence of APOE-ε4 genotype (*p*=0.07) (Table 2B). In the pool data, BNP values did not significantly differ in patients carrying or not cognitive impairment.

**Table 2:** Association between genotypes and BNP value (*A*) or percentage of cognitive impairment (*B*) in the study population.

A			
**SNPs**		**BNP (pg/mL)**	***p***
***P2X7R*** rs3751143	A carriers C carriers	702.0 ± 115.0 674.3 ± 167.0	0.892
***P2X7R*** **rs208294**	C carriers T carriers	626.2 ± 162.6 721.6 ± 103.4	0.621
***APOE*** **rs429358 rs7412**	ε4 carriers ε4 noncarriers	1289.9 ± 226.9 580.5 ± 90.2	0.004

*B*			
**SNPs**		**Cognitive impairment (% of patients)**	***p***
***P2X7R*** rs3751143	A carriers C carriers	49 49	0.892
***P2X7R*** **rs208294**	C carriers T carriers	46 50	0.621
***APOE*** **rs429358 rs7412**	ε4 carriers ε4 noncarriers	63 37	0.07

Data are mean±SEM; *p* values are from ANOVA.

### Follow-up

Thirty-six months after discharge from hospital, 93 (47%) patients were still alive. As expected, at the admission they were younger (83 ± 1 *vs* 87 ± 1 years, *p*<0.0001), presented a lower number of comorbidities (Charlson index 7.4 ± 0.3 *vs* 8.1 ± 0.2, *p*<0.05) and cognitive impairment was less represented (28 *vs* 13%, *p*<0.0001); no difference was observed according to gender.

**Figure 1.**
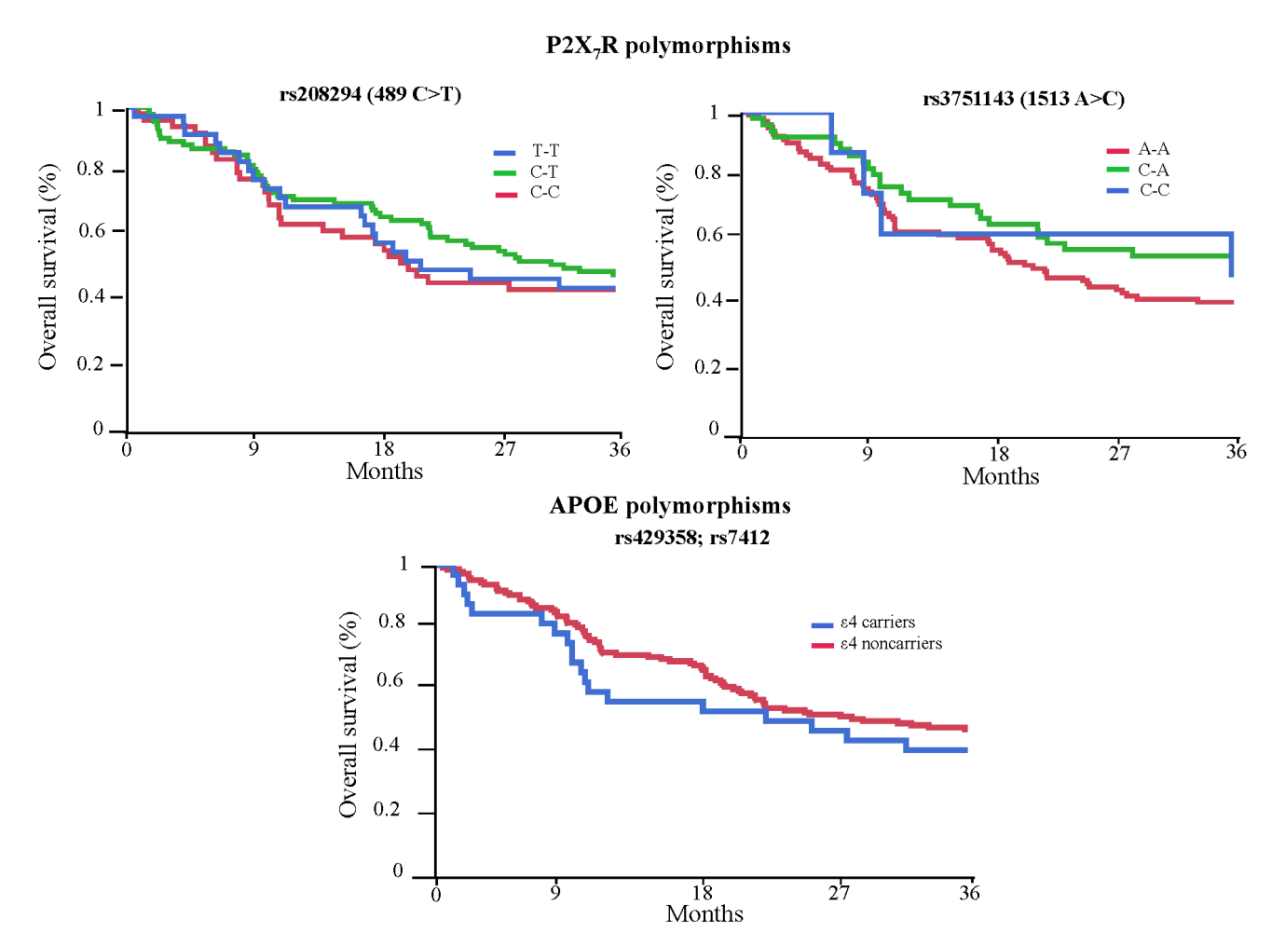
Overall survival curves of all the patients according to genetic profiles of the studied polymorphisms Survival curves were calculated with the Kaplan Meier method.

No significant association between polymorphisms of interest and overall survival (OS) was found (Fig. 1). This result was confirmed even when the analysis was run comparing carriers and non carriers of the mutant allele for P2X_7_R and APOE genes polymorphisms. However, when the whole cohort was divided according to BNP median value during hospitalization (281 pg/mL), a lower OS was observed in those having higher values (*p*=0.002). After running a proportional hazards regression analysis, the relationship between BNP values and reduced OS still remained significant (*p*=0.03) (Suppl Table B). Likewise, the presence of cognitive impairment was significantly associated with higher mortality rate (*p*<0.001).

## DISCUSSION

The main finding of the present study is a negative one: even though P2X_7_R (rs208294 and rs3751143) and APOE (rs429358 and rs7412) polymorphisms have been recognized as potential determinants of unfavorable prognosis in several and heterogeneous morbid conditions [29, 30], they were not independently associated to three-year post-discharge mortality in a cohort of older patients, admitted at emergency room for HF. Instead, our observation that higher BNP values and impairment in cognitive status tracked the poorest outcome deserves attention. Lastly, whilst P2X_7_R polymorphisms were linked neither to HF and cognitive decline, APOE-ε4 carriers showed significantly higher BNP values, indicative of a cardiac dysfunction.

Other groups have previously evaluated the impact of SNPs in P2X_7_R gene on clinical prognostic markers and survival in different clinical contexts from ours, like in oncological diseases, reporting a neutral effect, although significant interactions with other genes are reported [31, 32]. APOE polymorphisms are, instead, according to a vast literature, among the genes affecting life span; notably, APOE-ε4 allele is associated with an increased mortality risk during adulthood [33]. Controversy exists whether this relationship is still valid at the oldest ages; our results are in line with those supporting the notion of a reduced effect of the ‘risk’ ApoE-ε4 allele in elderly individuals [34]; however, in our population ApoE-ε4 homozygosity was not represented (likely for the limited size of our cohort) and several other causes could compete as mortality risks.

On the other hand, we confirmed the prognostic value of natriuretic peptides in elderly hospitalized patients for HF, in terms of post-discharge mortality [35]. We found also a consistent association between cognitive impairment and poor outcome; these data confirm those of other authors obtained in aging patients [36], even affected by HF [37].

An intriguing, novel observation is that APOE-ε4carriers present significantly higher BNP concentrations than non carriers. Previous reports suggested a positive association between systolic dysfunction and APOE-ε4 genotype, although it was more pronounced for homozygous than heterozygous carriers; odds ratio increased when the study population was restricted to subjects aged 65 years or older, and did not significantly change when controlling for different causes of ventricular dysfunction [38]. Moreover, Van der Cammen *et al*. showed that, in patients affected by Alzhemeir’s disease, the risk of having electrocardiographic abnormalities indicative of left ventricular dysfunction was over seven fold increased [39]. Our results endorse the hypothesis of an effect of APOE-ε4 genotype on ventricular dysfunction, at least as estimated by BNP values. Besides, it is well documented that BNP concentrations have a prognostic role in the natural history of HF [40], as shown by the impact on mortality rate in our cohort.

Several limitations need to be highlighted in the present study. From a clinical/methodologic view point, echocardiographic data in the whole cohort would have better characterized the HF spectrum, as well as a complete multifunctional evaluation in the cognitive function would have allowed a more refined diagnosis of the cognitive decline and its causes. Besides, likely due to the limited number of patients, APOE-ε4 homozygous carriers were not represented, so that any recessive effect could be explored. The main strength is to have further investigated the role of SNPs potentially involved in the pathogenesis of HF and cognitive disorders, highly prevalent in a geriatric population, as determinants of all-cause mortality. Furthermore, our work shows for the first time a positive association between BNP values, surrogate/marker of ventricular dysfunction, and ApoE-ε4 genotype, which is broadly known to affect cardiovascular morbidity; such preliminary observation requires, obviously, to be confirmed by others.

In conclusion, in our cohort of frail elderly individuals common P2X_7_R and APOE polymorphisms do not influence long-term all-cause mortality. Further studies involving larger population will be able to answer the question whether major and impactful events could have blunted the effect of such polymorphisms on clinical outcome, as well as to better characterize the relationship between heart failure and APOE gene polymorphisms.

**Suppl Table A:** Genotypes, allele frequencies and Hardy-Weinberg Equilibrium (HWE) of the studied polymorphisms

SNP	Genotype	N	Allele	N	%	HWE ( *p*)
***P2X7R*** **rs208294**	T-T C-T C-C	38 100 52	C T	152 138	0.90 0.10	0.42
***P2X7R*** **rs3751143**	A-A C-A C-C	111 55 8	A C	166 63	0.72 0.28	0.72
***APOE*** **rs7412**	C-C C-T	181 17	C T	198 17	0.92 0.08	0.53
***APOE*** **rs429358**	T-T C-T	157 32	C T	32 189	0.14 0.86	0.20

**Suppl Table B:** Proportional hazards regression analysis showing BNP impact on reduced overall survival

	Β coefficient	*p*
**BNP**	0.0003	0.032
**Age**	0.046	0.084
**Sex (f)**	-0.162	0.340
**Charlson comorbidity index**	0.079	0.257
